# Divergence in gene expression within and between two closely related flycatcher species

**DOI:** 10.1111/mec.13596

**Published:** 2016-04-02

**Authors:** Severin Uebbing, Axel Künstner, Hannu Mäkinen, Niclas Backström, Paulina Bolivar, Reto Burri, Ludovic Dutoit, Carina F. Mugal, Alexander Nater, Bronwen Aken, Paul Flicek, Fergal J. Martin, Stephen M. J. Searle, Hans Ellegren

**Affiliations:** ^1^ Department of Evolutionary BiologyEvolutionary Biology CentreUppsala UniversityNorbyvägen 18DSE‐752 36 UppsalaSweden; ^2^ European Molecular Biology LaboratoryEuropean Bioinformatics InstituteWellcome Trust Genome CampusHinxtonCambridgeUK; ^3^ Wellcome Trust Sanger InstituteWellcome Trust Genome CampusHinxtonCambridgeUK; ^4^ Present address: Lübeck Institute of Experimental DermatologyUniversity of LübeckLübeckGermany; ^5^ Present address: Guest Group Evolutionary GenomicsMax Planck Institute of Evolutionary BiologyPlönGermany; ^6^ Present address: Department of BiologyUniversity of TurkuTurkuFinland

**Keywords:** collared flycatcher, *Ficedula*, gene regulation, pied flycatcher, speciation, transcriptomics

## Abstract

Relatively little is known about the character of gene expression evolution as species diverge. It is for instance unclear if gene expression generally evolves in a clock‐like manner (by stabilizing selection or neutral evolution) or if there are frequent episodes of directional selection. To gain insights into the evolutionary divergence of gene expression, we sequenced and compared the transcriptomes of multiple organs from population samples of collared (*Ficedula albicollis*) and pied flycatchers (*F. hypoleuca*), two species which diverged less than one million years ago. Ordination analysis separated samples by organ rather than by species. Organs differed in their degrees of expression variance within species and expression divergence between species. Variance was negatively correlated with expression breadth and protein interactivity, suggesting that pleiotropic constraints reduce gene expression variance within species. Variance was correlated with between‐species divergence, consistent with a pattern expected from stabilizing selection and neutral evolution. Using an expression *P*_ST_ approach, we identified genes differentially expressed between species and found 16 genes uniquely expressed in one of the species. For one of these, *DPP7*, uniquely expressed in collared flycatcher, the absence of expression in pied flycatcher could be associated with a ≈20‐kb deletion including 11 of 13 exons. This study of a young vertebrate speciation model system expands our knowledge of how gene expression evolves as natural populations become reproductively isolated.

## Introduction

It seems increasingly clear that changes in gene expression provide key steps in the molecular basis of adaptation (King & Wilson 1975; Wray *et al*. 2003; Hoekstra & Coyne 2007; López‐Maury *et al*. 2008; Romero *et al*. 2012; Pardo‐Diaz *et al*. 2015). There are many examples of differential gene expression causing adaptive phenotypic changes (Abzhanov *et al*. 2006; Chan *et al*. 2010; McBride *et al*. 2014). It has also been suggested that changes in gene expression should commonly lead to speciation (Haerty & Singh 2006), although concrete examples for such a causative relationship between expression divergence and reproductive isolation are rare (Kradolfer *et al*. 2013; Thomae *et al*. 2013; Chung *et al*. 2014; Dion‐Côté *et al*. 2014). Moreover, phenotypic plasticity rather than genetically determined changes in gene expression might explain observed differences in gene expression between populations or species in different environments (Cheviron *et al*. 2008; Aguilar *et al*. 2010; Morris *et al*. 2014). At the same time, plasticity can facilitate local adaptation by enabling initial survival in new environments, which may or may not be followed by genetically determined changes in gene expression (López‐Maury *et al*. 2008; Pavey *et al*. 2010; Côté *et al*. 2014; Meier *et al*. 2014). Either way, genetically mediated regulatory changes leading to diverged gene expression patterns are expected to accumulate as species diverge.

Relatively little is known about the character of gene expression evolution when species split and diverge. It has been suggested that gene expression may generally evolve under a combination of stabilizing selection and neutral evolution, a conclusion drawn from the observation that within‐ and between‐population expression variances have been found to be correlated in some mammalian species (Brawand *et al*. 2011; Gilad 2012; Romero *et al*. 2012). Genes with deviating expression patterns might represent cases of either directional or balancing selection (Whitehead & Crawford 2006; Romero *et al*. 2012). More specifically, genes may show expression patterns consistent with (i) stabilizing selection (small expression variance within and between species), (ii) neutral evolution (large expression variance within and between species), (iii) directional selection (small expression variance within and large variance between species) or (iv) balancing selection (small expression variance between‐species and large variance within‐species expression variance) (Whitehead & Crawford 2006). Meanwhile, recent findings suggest that pleiotropic interactions, such as approximated by the number of protein–protein interactions, constrain gene expression evolution in the early stages of divergence (Papakostas *et al*. 2014).

Collared flycatcher (*Ficedula albicollis*) and pied flycatcher (*F. hypoleuca*) are two passerine bird species breeding in Europe that diverged no more than one million years ago (Backstrom *et al*. 2013; Nadachowska‐Brzyska *et al*. 2013; Nater *et al*. 2015). Genome sequencing and assembly followed by whole‐genome resequencing of both species has revealed moderate levels of genetic differentiation with a mean genomewide *F*_ST_ of 0.26 (Ellegren *et al*. 2012; Burri *et al*. 2015). The mean pairwise sequence divergence between allopatric populations of the two species (*d*_xy_, 0.0048) was found to be only slightly higher than the mean pairwise nucleotide diversity in intraspecific comparisons (collared flycatcher: 0.0040; pied flycatcher: 0.0032), demonstrating that most variation is found within rather than between species (Ellegren *et al*. 2012; Burri *et al*. 2015). The genomic landscape of differentiation is heterogeneous with distinct regions of increased differentiation ‘differentiation islands’, associated with reduced nucleotide diversity and spread over most chromosomes (Ellegren *et al*. 2012; Burri *et al*. 2015). These data on sequence diversity and divergence provide a useful background against which to contrast diversity and divergence in gene expression, making *Ficedula* flycatchers a good model for studying gene expression evolution in a young speciation system.

Although numerous gene expressions studies have been conducted in nonmodel and model organisms, only few studies have used multiple organ types to estimate expression diversity and divergence. However, such studies are especially called for because it is well known that gene expression patterns differ between organs or even between tissue and cell types within an organ (Alvarez *et al*. 2015). Here, we performed transcriptome sequencing of nine different organs in population samples of pied and collared flycatcher allowing us to collect a comprehensive catalog of gene expression variation at multiple levels of biological organization. We estimated gene expression diversity and divergence among organs within species, among individuals and between species. We then compared expression divergence to coding sequence and upstream sequence evolution and different measures of pleiotropy to investigate how different evolutionary forces affect divergence in gene expression. Finally, we searched for genes that were uniquely expressed in one species, and found evidence for a large deletion causing a marked difference in gene expression in an early stage of speciation.

## Material and methods

### Sampling and sequencing

Data generation for collared flycatcher samples (four adult females and five adult males, and eight embryos) has been described earlier (Uebbing *et al*. 2013). In addition, we collected 10 unrelated adult (five females and five males) and eight embryos of pied flycatchers in Uppsala, Sweden, which were treated in the same way. Adult birds of the two species were sampled at the same time point in the breeding cycle and were killed by decapitation and immediately dissected. Brain, kidney, liver, lung, muscle, ovary, skin and testis organs were collected and immediately stored in RNAlater (Qiagen). We use the term ‘organ’ instead of the perhaps more commonly used term ‘tissue’ because organs typically contain several types of tissues, including vascular, neural and connective tissues. Embryos were matched for age by collection of eggs shortly after laying and before brooding had started, followed by incubation for 7 days after which samples were taken. Sampling was conducted according to permissions and rules of the Swedish ethics committee for wild animals (2007/C319—Uppsala Djurförsöksetiska nämnd).

Total RNA was extracted and yielded sufficient concentrations of RNA with integrity numbers higher than eight. RNA was poly‐A enriched, reverse transcribed into cDNA, indexed individually per sample and sequenced on an Illumina Genome Analyzer IIx for 100 cycles. Sequencing produced 484.8 and 527.9 million paired‐end reads for collared flycatchers and pied flycatchers, respectively.

### Genome annotation

The annotation of genes and genome features was carried out using the Ensembl gene annotation system (Curwen *et al*. 2004). The genome was repeat‐masked using a combination of the RepeatMasker ‘aves’ Repbase library and a custom repeat library generated with RepeatModeler (Morgulis *et al*. 2006). These repeats were used for gene annotation purposes. Additional repeats were generated using Dust (http://web.mit.edu/seven/src/ncbi/tools/dust.c) and TRF (Benson 1999).

Protein‐coding genes were annotated using a combination of the flycatcher RNA‐seq data and protein homology. RNA‐seq transcript models were generated by first mapping the reads to the genome using BWA (Li & Durbin 2009) and then locating exons and intron spanning reads. RNA‐seq models were validated by aligning protein existence level 1 and level 2 proteins from UniProt (UniProt Consortium 2015) onto the transcript and selecting only transcripts that had a match with at least 50% hit coverage and identity.

Homology models were generated by aligning the UniProt vertebrate protein set to the genome using GeneWise (Birney *et al*. 2004). In addition, the longest translations of each Ensembl chicken (*Gallus gallus*) and zebra finch (*Taeniopygia guttata*) protein‐coding gene were aligned to the genome using Exonerate (Slater & Birney 2005). The final set of genes was filtered to remove low‐quality and redundant models, with preference given to selecting a protein‐validated RNA‐seq model or a known bird protein model at each genomic position.

Noncoding gene models were generated by carrying out a blast (Altschul *et al*. 1990) of the mirbase (Kozomara & Griffiths‐Jones 2014) and Rfam (Burge *et al*. 2013) databases against the flycatcher genome. Pseudogenes were identified from single‐exon transcripts by identifying pseudogenic signals, such as a spliced version of the sequence located elsewhere in the genome.

For further information on generating the ensembl flycatcher annotation see Supporting information.

### Data preparation

All but three copies of duplicated paired‐end reads were discarded as those most likely stem from highly duplicated regions or represent PCR artefacts. We mapped reads onto the flycatcher genome build ficalb1.5 (Kawakami *et al*. 2014) using tophat v. 2.0.10 (Kim *et al*. 2013) and extracted FPKM normalized gene expression values for Ensembl flycatcher genes using cufflinks v. 2.1.1 (Trapnell *et al*. 2012). FPKM values were then further normalized using the procedure described in Hart *et al*. (2013), and genes were defined as being expressed using the cut‐off of 0.125 zFPKM suggested in that study. We also extracted raw read counts from these mappings for analyses of differential expression in edgeR.

### Statistical analyses

We calculated Euclidean distances from zFPKM expression values between all organ/individual combinations (i.e. sequencing libraries) and used nonmetric multidimensional scaling (NMDS) as implemented in the r package mass v. 7.3–26 (Venables & Ripley 2002) for ordination plotting. The number of used axes (three for collared flycatchers only, four for both flycatchers and both flycatchers with chicken) has been determined visually using scree plots. The scaling procedure was iterated until convergence. We used analysis of variance (anova) on NMDS axes values for samples with ‘organ’, ‘sex’, ‘species’ and their interactions as fixed effect factors. For comparison with chicken, we used three gene expression data sets of that species (NCBI Accession nos. PRJNA143627 (Brawand *et al*. 2011), PRJEB4677, PRJNA248570).

*P*_ST_ is a measure comparing within‐ and between‐population variance of a quantitative trait, in this case the gene expression level, and was calculated as in Antoniazza *et al*. (2010) using ‘sex’ as a random‐effects factor. Differentially expressed (DE) genes were determined using 1000 resampled replicates per organ, and *P*‐values were resampling corrected according to Phipson & Smyth (2010). *P*_ST_ estimates were compared with the results from tests for differential expression in edgeR (Robinson *et al*. 2010) using raw read counts and standard options (again with ‘sex’ as a random‐effects factor).

We tested for correlations using Spearman rank correlation and for differences between group means using Mann–Whitney *U*‐tests, if not noted otherwise. Analyses including multiple tests were Benjamini–Hochberg corrected. Expression specificity (τ) was calculated following Yanai *et al*. (2005). When analysing low and high expression variance genes, expression variance was controlled for expression level, gene length, GC content, organ, sex and species. Tests for enrichment of gene ontology terms were performed with the r bioconductor package goseq (Young *et al*. 2010). Significant GO terms were Bonferroni corrected for multiple testing. r scripts for calculating zFPKM, *P*_ST_ and τ are available online at https://github.com/severinEvo/gene_expression.

### Protein–protein interactions

Data for the number of protein–protein interactions of chicken genes were obtained from the funcoup v. 3.0 database (Schmitt *et al*. 2013); chicken is the only bird species for which genomewide interactivity data is available. Data were extracted for metabolic networks, protein complexes and signalling cascades and were limited to 9951 1:1 orthologs between chicken and flycatcher. We used interactions with an FBS score of at least 7.

### DNA sequence variation

Estimates of nucleotide diversity and distribution of sequence coverage were obtained from Burri *et al*. (2015) and were based on whole‐genome resequencing data from 79 individuals per species. *F*_ST_ between collared flycatcher and pied flycatcher in 2‐kb genomic regions upstream of the translation start site of each gene was estimated based on genotype likelihoods using angsd (Nielsen *et al*. 2012) and ngstools (Fumagalli *et al*. 2014). These 2‐kb windows served as proxies for the location of potential regulatory sites in the absence of annotations of regulatory sequences or transcription start sites in flycatchers. We retrieved 1:1:1 orthologous coding sequences of collared flycatcher, zebra finch and chicken from Ensembl release 73 (Flicek *et al*. 2014) and generated codon‐based alignments using prank v.130410 (Löytynoja & Goldman 2005). Misaligned columns according to the heads‐or‐tails (HoT) algorithm as implemented in guidance using default settings were discarded (Landan & Graur 2007; Penn *et al*. 2010). We estimated flycatcher lineage‐specific nonsynonymous (*d*_N_) and synonymous (*d*_S_) substitution rates using a free‐ratio model (one *d*_N_/*d*_S_ per branch) using codeml from paml v. 4.7 (Yang 2007). We excluded genes with *d*_S_ > 2 and *d*_N_/*d*_S_ > 3 because high estimates of *d*_S_ may indicate saturation in synonymous sites or alignment errors, which may produce unreliable *d*_N_/*d*_S_ estimates.

## Results

### Genome annotation identifies gene models

The integration of RNA‐seq data with the ensembl pipeline for annotation of genes in the collared flycatcher genome led to the identification of a total of 16 266 genes, including 37 genes from the mitochondrial genome. This is comparable to the amount found in the Ensembl annotations of other birds, that is chicken (Galgal4, 17 108 genes), duck (*Anas platyrhynchos*, BGI_duck_1.0, 16 450 genes), turkey (*Meleagris gallopavo*, melGal1, 15 002 genes) and zebra finch (taeGut3.2.4, 18 618 genes). The genes could be classified into 15 303 protein‐coding, 897 noncoding and 66 pseudogenes. RNA‐seq evidence was used in the creation of the majority of protein‐coding gene models (12 238), while some of the protein‐coding models were derived solely from alignments of homologous proteins from uniprot and ensembl. The 897 noncoding models were generated from blast alignments of mirbase and rfam databases. The 66 pseudo‐genes were identified using the ensembl pseudo‐gene pipeline, which looks for pseudogenic signals in single‐exon gene models. All annotations were made available as part of ensembl release 73 and are viewable in the ensembl genome browser (http://www.ensembl.org/). A supplementary set of organ‐specific RNA‐seq models spanning nine organs and their associated bam files are available as additional tracks in the browser.

These data plus similarly generated data from pied flycatcher (with 8–10 individuals sequenced of each species) resulted in an average of 7.1 million reads per individual for each organ and led to the quantification of expression levels of 12 052 (liver, pied flycatcher) to 13 871 (ovary, collared flycatcher) genes per organ (Table S1, Supporting information). Although 65.5% of filtered RNA‐seq reads mapped to the collared flycatcher reference genome, only 7.1% of the reads mapped onto annotated gene models.

### Ordination separates samples according to organ

**Figure 1:**
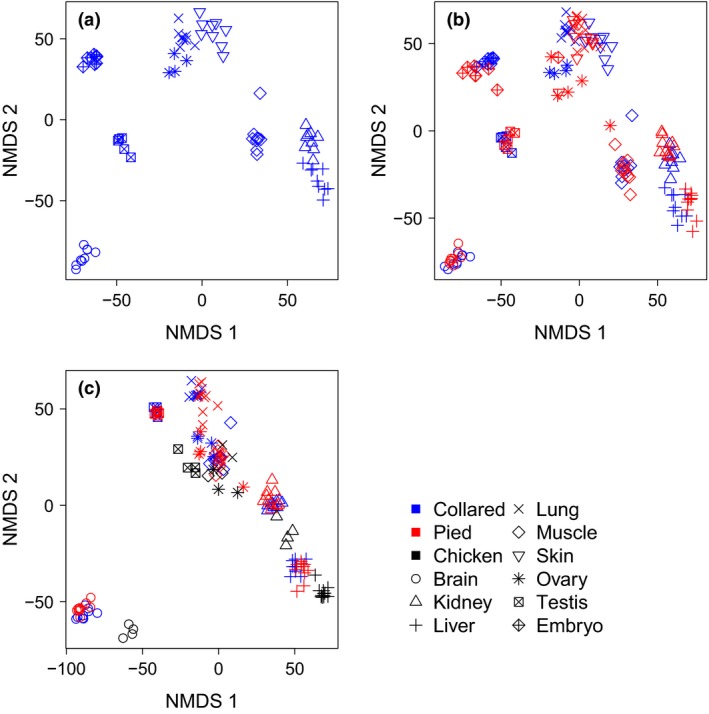
Ordination of gene expression data from (a) collared flycatcher samples, (b) collared and pied flycatcher samples and (c) flycatcher and chicken samples using NMDS. Note that the actual values of the axes are arbitrary and that only relative distances are of importance.

We used nonmetric multidimensional scaling (NMDS) for ordination analysis of all samples (i.e. all individual/organ combination), initially focusing on collared flycatcher. All organs resolved into separate clusters, demonstrating unique and organ‐specific transcriptome profiles (Fig. 1a; Table S2, Supporting information). Lung, ovary and skin organs grouped very close to each other, as did kidney and liver.

**Figure 2:**
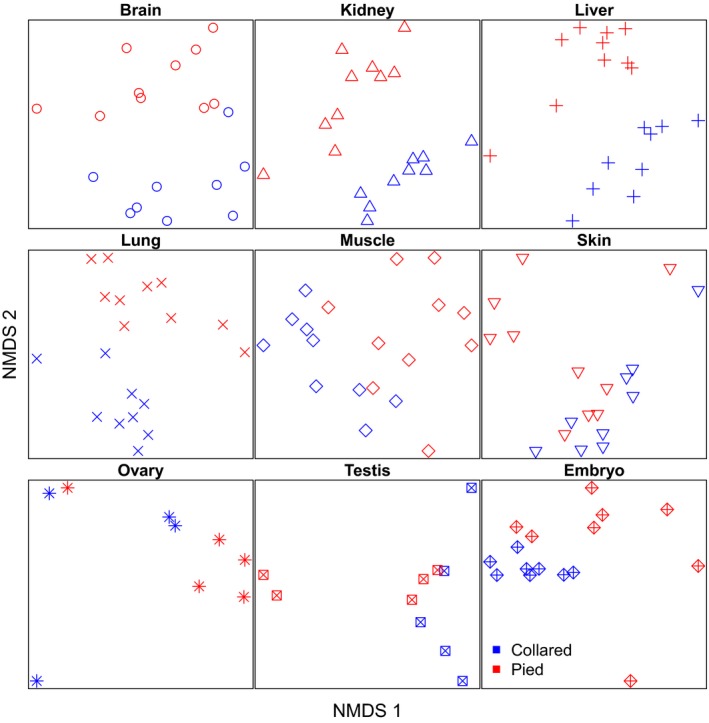
Separate NMDS ordination plots of all analysed tissues. Symbols are as in Fig. 1 for consistency.

Addition of pied flycatcher to the NMDS showed that samples clustered mainly by organ and not by species (Fig. 1b), with substantial amount of overlap between samples from the two species. This was confirmed using anovas, showing that organ identity had consistently strong effects on the data. The effect of species identity was only significant on NMDS axis 3, while sex showed significant effects already on axis 2 (Table S3, Supporting information). However, when separate ordination plots were made for each organ, species tended to resolve within several of the organs (Fig. 2). Importantly, five of the nine organs resolved fully according to species and all but one (ovary) showed a tendency to do so. There was thus overall a very weak but detectable signal of interspecies difference in gene expression (Fig. 2).

Adding expression data from the distantly related chicken put more emphasis on the phylogenetic aspect of the data in a three‐species ordination plot. Collared and pied flycatcher samples clustered closely together and organ differences caused most variance. The chicken data clustered according to the respective organs but more distantly from flycatcher samples (Fig. 1c). An anova showed consistently stronger effects for organ than species, while sex did not explain much of the observed variance (Table S4, Supporting information).

### Differences in gene expression levels among individuals and species

Expression variance among individuals varied considerably between organs and was largest in ovary and smallest in testis (Fig. S1, Supporting information). Expression variance for individual genes was correlated among organs (Spearman ρ ranging from 0.148 between liver and ovary, to 0.476 between lung and skin, both in pied flycatcher, *P *<* *2.2 × 10^−16^ for all comparisons; Table S5, Supporting information). The genes in the lower 10th percentile of expression variance were enriched for gene ontology (GO) terms related to intracell signalling functions and response to external stimuli (Table S6, Supporting information). Terms enriched in the upper 10th percentile included terms related to extra‐cellular space and hormone activity as well as muscular functions (Table S6, Supporting information). Genes in the high variance group were more often (23 of genes) lacking GO annotations than genes in the low variance group (18%; Fisher's exact test, *P *=* *0.0099).

Within‐species expression variance was correlated with the difference in log_2_ mean expression level between species for all organs [Spearman ρ ranged between 0.068 (liver) and 0.311 (skin), *P *< 10^−12^ in all cases]. We treated expression level as a quantitative trait and used *P*_ST_ to measure expression differentiation between species relative to within‐species expression variance and identified differentially expressed (DE) genes by resampling. Organs differed in the number of DE genes, from 2.4% in brain to 26.7% in liver (average 12.8% over all nonreproductive organs, Table 1). Ovary and testis had smaller sample sizes (five birds per organ) and thus lower power to detect DE genes; 0.5% and 1.2% DE genes were identified in these organs, respectively. The power to detect differentially expressed genes is lower for genes with low expression levels, but any employed cut‐off is arbitrary and does in this case not change the relative proportions of differentially expressed genes among organs (Table S7, Supporting Information). Where possible, we used the distribution of *P*_ST_ values instead of a contrasted set of significant vs. insignificant genes to avoid any such issue. *P*_ST_ was independent of expression level (Spearman ρ ranging from −0.013 in embryo to 0.020 in ovary, *P *>* *0.1 in all organs). High *P*_ST_ values could in theory be driven by small within‐species variance only, large between‐species variance only or a combination of both. Inspection of variance components showed similar distributions among DE genes and the rest of the data set (Fig. S2, Supporting information), indicating that *P*_ST_ was not generally driven by within‐ or between‐species variance alone. The proportion of differentially expressed genes did not differ between the Z chromosome (on average 9.7% over organs) and autosomes (9.2%; χ^2^ = 0.0497, *P* =0.82). Similarly, the proportion of differentially expressed genes was independent of chromosome size (Fig. S3, Supporting information). A limited number of gene ontology (GO) categories were enriched among *P*_ST_ DE genes and included mitochondrion, structural constituent of ribosome, translation and ribonucleoprotein complex (Table S8, Supporting information).

**Table 1:** Numbers of differentially expressed genes between collared and pied flycatcher

Organ	DE genes (*P*_ST_)	DE genes (edgeR)	Overlap^a^
Brain	197 (2.4%)	87 (0.8%)	33.3%
Kidney	1221 (14.4%)	721 (6.0%)	64.4%
Liver	1779 (26.7%)	521 (5.4%)	68.1%
Lung	1557 (18.4%)	285 (1.9%)	54.4%
Muscle	483 (6.8%)	307 (3.0%)	38.8%
Skin	906 (10.2%)	807 (6.0%)	46.3%
Embryo	860 (10.1%)	149 (1.2%)	65.1%
Mean	1000 (12.7%)	411 (3.5%)	52.9%
Ovary	38 (0.5%)	67 (0.5%)	0.0%
Testis	113 (1.2%)	90 (0.8%)	13.3%
Mean	795 (10.1%)	337 (2.9%)	42.6%

a Proportion of genes detected by both *P*_ST_ and edgeR relative to all genes detected by edgeR.

We compared the *P*_ST_ approach to an established protocol for detecting differentially expressed genes, edgeR. As expected from a correspondence between the two approaches, edgeR *P*‐values (low values meaning high divergence) showed negative correlations with *P*_ST_ (high values meaning high divergence; Spearman ρ ranging from −0.102 in ovary to −0.784 in kidney, *P *<* *2.2 × 10^−16^ in all cases). On average, 52.9% of the genes identified to be differentially expressed by edgeR were detected as *P*_ST_ DE genes as well (Table 1). Similar to *P*_ST_ DE genes, differentially expressed genes identified with edgeR were enriched for only a limited number of rather general GO categories (Table S8, Supporting information), including mitochondrial enzymatic functions in kidney and liver.

### Divergence in gene expression does not relate to sequence divergence

To test whether divergence in gene expression was related to divergence in potential regulatory sequences, and assuming *cis*‐regulatory effects, we estimated *F*_ST_ in the 2‐kb region upstream of the translation start site of each gene. *P*_ST_ was not correlated with this estimate of *F*_ST_ in any organ (Spearman rank correlations, *P *> 0.1 in all organs). Similarly, genes situated in genomic regions with high *F*_ST_ (variable cut‐offs between 0.3 and 0.8) did neither show significantly higher *P*_ST_ (Mann–Whitney *U*‐tests, *P *> 0.05 in 49 of 54 cases) nor an increased number of *P*_ST_ DE genes compared to genomic background levels (χ^2^ tests, *P *> 0.1 in all cases). However, the distribution of *P*_ST_ values was strongly skewed towards zero, suggesting that there might be little signal for a correlation. We therefore repeated the tests using lower *P*_ST_ cut‐offs (varying between 0.05 and 0.3), but found no evidence for significant correlations.

To test whether gene expression divergence was related to the divergence of protein sequences, we estimated *d*_N_/*d*_S_ in the lineage leading towards flycatcher from three‐species alignments of flycatcher, zebra finch and chicken orthologs. Correlations between *P*_ST_ and *d*_N_/*d*_S_ were not significant for most organs, with the exception of skin (ρ = −0.084, *P *=* *6.4 × 10^−7^) and embryo (ρ = −0.076, *P *=* *6.6 × 10^−^) (Table S9, Supporting information). However, closer inspection of the *P*_ST_ variance components showed consistent positive correlations between the within‐species variance component and *d*_N_/*d*_S_ [range: ρ = 0.071 in testis (*P *=* *4.2 × 10^−6^) to 0.208 in lung (*P *<* *2.2 × 10^−16^)], while correlations between *d*_N_/*d*_S_ and between‐species variance were weak or absent (Table S9, Supporting information).

### Pleiotropy influences gene expression variance, but not its evolution

The number of protein–protein interactions (interactivity) a gene is involved in was positively correlated with *P*_ST_ (Table 2). This resulted from strong negative correlations between the number of interactions and *P*_ST_'s within‐species variance component (range: ρ = −0.131 for signalling cascade interactions in brain to −0.365 for metabolic chain interactions in skin, *P *< 2.2 × 10^−16^ in all cases), while the relationships between the number of interactions and the between‐species variance component did not show any clear pattern (Table 2). There was a general tendency for correlations to be strongest for metabolic chains, intermediate for protein complexes and weakest for signalling cascades (Table 2).

**Table 2:** Spearman rank correlations (ρ) of *P*_ST_ and its variance components with three different types of protein–protein interactions

	*P*_ST_					
	Metabolic chains		Protein complexes		Signalling cascades	
	ρ	*P* value	ρ	*P* value	ρ	*P* value
Brain	0.023	0.090	0.034	0.012	0.038	0.0043
Kidney	0.049	2.1 × 10^−4^	0.045	6.5 × 10^−4^	0.007	0.62
Liver	0.140	<2.2 × 10^−16^	0.125	<2.2 × 10^−16^	0.062	1.6 × 10^−5^
Lung	0.118	<2.2 × 10^−16^	0.127	<2.2 × 10^−16^	0.065	4.4 × 10^−7^
Muscle	0.007	0.62	0.007	0.62	0.009	0.58
Skin	0.034	0.0082	0.032	0.013	0.026	0.043
Ovary	−0.025	0.053	−0.040	0.0024	−0.009	0.53
Testis	0.045	3.1 × 10^−4^	0.039	0.0016	0.010	0.47
Embryo	0.082	1.1 × 10^−10^	0.057	1.0 × 10^−5^	0.081	1.8 × 10^−10^
	Between−species variance (σ_b_)					
Brain	0.002	0.90	0.004	0.83	0.028	0.065
Kidney	0.025	0.091	0.024	0.10	−0.011	0.47
Liver	0.082	2.0 × 10^−8^	0.076	1.4 × 10^−7^	0.028	0.085
Lung	−0.092	1.1 × 10^−12^	−0.115	<2.2 × 10^−16^	−0.018	0.19
Muscle	−0.026	0.091	−0.021	0.17	−0.022	0.16
Skin	−0.001	0.93	0.005	0.77	0.028	0.058
Ovary	−0.022	0.13	−0.038	0.0080	0.003	0.87
Testis	0.053	4.0 × 10^−5^	0.044	8.9 × 10^−4^	0.011	0.43
Embryo	0.041	0.0031	0.019	0.17	0.031	0.036
	Within−species variance (σ_w_)					
Brain	−0.170	<2.2 × 10^−16^	−0.156	<2.2 × 10^−16^	−0.131	<2.2 × 10^−16^
Kidney	−0.284	<2.2 × 10^−16^	−0.239	<2.2 × 10^−16^	−0.167	<2.2 × 10^−16^
Liver	−0.304	<2.2 × 10^−16^	−0.277	<2.2 × 10^−16^	−0.162	<2.2 × 10^−16^
Lung	−0.254	<2.2 × 10^−16^	−0.181	<2.2 × 10^−16^	−0.191	<2.2 × 10^−16^
Muscle	−0.313	<2.2 × 10^−16^	−0.281	<2.2 × 10^−16^	−0.167	<2.2 × 10^−16^
Skin	−0.365	<2.2 × 10^−16^	−0.304	<2.2 × 10^−16^	−0.227	<2.2 × 10^−16^
Ovary	−0.234	<2.2 × 10^−16^	−0.181	<2.2 × 10^−16^	−0.175	<2.2 × 10^−16^
Testis	−0.268	<2.2 × 10^−16^	−0.223	<2.2 × 10^−16^	−0.154	<2.2 × 10^−16^
Embryo	−0.321	<2.2 × 10^−16^	−0.277	<2.2 × 10^−16^	−0.202	<2.2 × 10^−16^

Organ specificity of expression (τ) and *P*_ST_ showed either no or only weak correlations (Table 3), and the same applied to correlations between τ and the between‐species variance component. In contrast, τ and the within‐species variance component were positively correlated in all organs but brain (range: ρ = 0.082 in pied testis, *P* = 3.6 × 10^−15^ to 0.367 in pied skin, *P *< 2.2 × 10^−16)^. These two observations—negative correlations between interactivity and within‐species variance, and positive correlations between organ specificity and within‐species variance—suggest that pleiotropic constraints reduce gene expression variance within but not between species.

**Table 3:** Spearman rank correlations (ρ) of *P*_ST_ and its variance components with expression specificity (τ)

Organ	*P*_ST_			
	Collared flycatcher		Pied flycatcher	
	ρ	*P* value	ρ	*P* value
Brain	0.042	0.0019	0.037	0.0070
Kidney	0.013	0.37	0.014	0.35
Liver	−0.008	0.60	0.014	0.37
Lung	−0.087	3.2 × 10^−11^	−0.065	8.6 × 10^−7^
Muscle	0.048	9.4 × 10^−4^	0.055	1.6 × 10^−4^
Skin	0.019	0.18	0.011	0.39
Ovary	−0.082	2.5 × 10^−10^	−0.034	0.012
Testis	0.027	0.036	0.002	0.86
Embryo	−0.030	0.028	−0.044	9.4 × 10^−4^
	Between−species variance (σ_b_)			
Brain	0.048	5.5 × 10^−5^	0.048	5.5 × 10^−5^
Kidney	0.019	0.12	0.029	0.016
Liver	0.023	0.090	0.044	8.0 × 10^−4^
Lung	0.028	0.021	0.013	0.30
Muscle	0.063	1.9 × 10^−6^	0.061	2.4 × 10^−6^
Skin	0.048	2.9 × 10^−5^	0.035	0.0025
Ovary	−0.094	6.1 × 10^−16^	−0.055	4.6 × 10^−6^
Testis	0.011	0.35	−0.010	0.38
Embryo	−0.008	0.49	−0.024	0.041
	Within−species variance (σ_w_)			
Brain	0.002	0.87	−0.002	0.87
Kidney	0.209	<2.2 × 10^−16^	0.204	<2.2 × 10^−16^
Liver	0.123	<2.2 × 10^−16^	0.126	<2.2 × 10^−16^
Lung	0.308	<2.2 × 10^−16^	0.311	<2.2 × 10^−16^
Muscle	0.185	<2.2 × 10^−16^	0.169	<2.2 × 10^−16^
Skin	0.351	<2.2 × 10^−16^	0.367	<2.2 × 10^−16^
Ovary	0.265	<2.2 × 10^−16^	0.270	<2.2 × 10^−16^
Testis	0.089	<2.2 × 10^−16^	0.082	<2.2 × 10^−16^
Embryo	0.295	<2.2 × 10^−16^	0.285	<2.2 × 10^−16^

The lack of a correlation between *P*_ST_, or its between‐species component, and proxies for pleiotropy could potentially be due to that the two flycatcher species are too closely related and have not diverged enough in their expression patterns (as was indicated by weak species effects in the NMDS anova analysis; Table S3, Supporting Information). We therefore analysed the relationship between proxies for pleiotropy and *P*_ST_ between collared flycatcher and chicken. This reproduced the patterns seen between the two flycatcher species: strong correlations of organ specificity and protein interactivity with the within‐species variance component but none or weak correlations with *P*_ST_ or between‐species variance (Table S10, Supporting Information).

### Some genes are specifically expressed in one species

**Figure 3:**
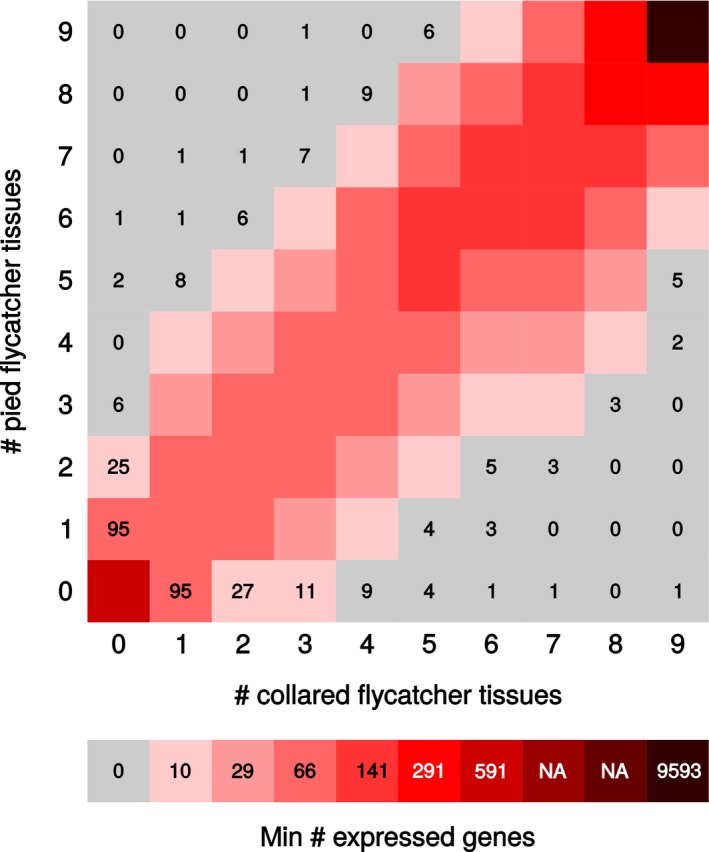
Heat map showing the number of genes detected as expressed in different tissues in collared and pied flycatcher. A large proportion of all genes (62.9%) are expressed in all tissues of both species. The leftmost column and the bottommost row show genes uniquely expressed in pied and collared flycatcher, respectively, with exact numbers given.

Between 50% and 70% of genes active in any organ were expressed in all individuals of both species. Not surprisingly, these typically represented the set of genes with highest expression levels per organ (Fig. S4, Supporting information). Genes with lower expression level remained undetected in some individuals, with genes showing the lowest level sometimes seen in one or only a few individuals as expected for stochastic reasons when expression levels are close to the cut‐off. As a likely consequence, most of the 1069 (embryo) to 1452 (ovary) genes unique to one of the species were lowly expressed. The precise numbers of genes considered to be unique to one species are sensitive to the definition of the presence of gene expression (see Material and methods). Importantly, the absence of detection in a species does not necessarily imply the absence of expression in that species. To reduce the influence of stochasticity in addressing absence/presence differences between species, we focused on genes that were expressed in at least five individuals of one species but completely absent from all organs in all individuals of the other species. Using these criteria, we found five genes to be exclusively expressed in collared flycatcher and 11 genes exclusively expressed in pied flycatcher (Fig. 3; Tables S11, S12, Supporting information).

**Figure 4:**
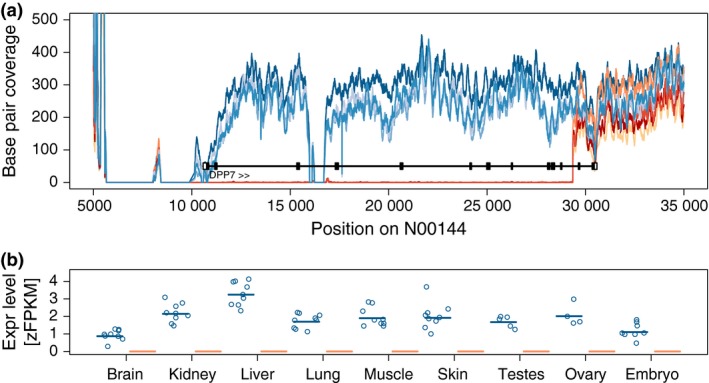
The first 11 of 13 exons of the *DPP7* gene correspond to a genomic deletion in pied flycatchers. (a) Genomic resequencing coverage for scaffold N00144 in collared (different shades of blue for different populations) and pied flycatchers (different shades of red for different populations). The black vertical line shows the location of the gene with filled boxes corresponding to coding sequences and open boxes to untranslated regions. Regions without coverage in any sample correspond to gaps in the genome assembly. (b) Expression level (zFPKM) of *DPP7* in different tissues of collared (black) and pied flycatcher (red). Circles show measures in individual birds while the vertical line depicts the tissue median. Expression in pied flycatchers was completely absent.

Genes that are exclusively expressed in one of the two species could potentially represent candidates for discrete genetic differences such as pseudogenization via structural changes in coding sequences or premature stop codons that lead to loss of transcription. To test for structural variation, we used whole‐genome resequencing data from 79 individuals of both species, with each individual sequenced at approximately 15× genome coverage (Burri *et al*. 2015), and screened the 16 species‐specific genes for clear differences in sequence coverage. We found one such case in the dipeptidyl‐peptidase 7 gene (*DPP7*). This gene is likely to be nonfunctional in pied flycatchers as a ≈ 20‐kb deletion is suggested by a complete lack of sequence coverage in a genomic region including the first 11 of 13 exons of the gene in this species (Fig. 4). The deletion appeared fixed in pied flycatchers as all 79 resequenced individuals had zero coverage in this region, whereas the sequence was present in all 79 resequenced collared flycatchers.

## Discussion

This study analysed expression profiles in multiple organs of population samples from two closely related bird species. Most organs showed unique transcriptome profiles within species. In an ordination analysis, some organs clustered more densely than others and most separated well from each other. When analysing both species, samples clustered primarily by organ and not by species as one would expect given that the evolutionary age of most organs is far older than that of the species. This was confirmed by an anova, which showed consistently stronger effects for organ than for species. Adding samples from the distantly related chicken further emphasized this pattern. These results are in line with a number of other studies showing a generally stronger signal for organ identity than species (Chan *et al*. 2009; Brawand *et al*. 2011; Gilad & Mizrahi‐Man 2015). Our sequencing design was confounded (one species/organ combination per Illumina lane) which is suboptimal (within‐organ x species effects cannot meaningfully be compared with between‐species or between‐organ effects) but leaves the comparison of organ‐ versus species‐specific effects unaffected by technical biases. Furthermore, the fact that chicken data obtained from other studies followed the trends observed in our data lent further credit to the observation of increased spatial distance in the NMDS with increased phylogenetic distance. In this context, we note that the zFPKM normalization procedure (Hart *et al*. 2013) was crucial for inclusion of the chicken data.

Although the amount of expression variance differed among organs, the expression variance of individual genes correlated among organs. Genes with low variance were enriched for a limited set of related GO categories showing involvement in basic cell signalling functions while genes with large expression variance showed enrichment of involvement in extracellular functions like blood hormone signalling, transporter activity and cytoskeleton‐related functions. High variance genes lacked GO annotations more often than low variance genes. This may suggest that some high variance genes are young genes or genes that have undergone rapid evolution. Both cases represent situations that may prevent annotation via homology.

To investigate divergence in expression levels, we treated gene expression as a quantitative trait and used *P*_ST_ (Roberge *et al*. 2007; Leinonen *et al*. 2013) to quantify expression divergence. This metric should be useful for detecting directional selection for gene expression as small within‐species variance in combination with large between‐species variance has been suggested to be indicative of directional selection in the evolution of gene expression (Wittkopp *et al*. 2008). We required a gene to be expressed in all samples of a tissue to include it into the test. We observed that a more stringent cut‐off did not change results qualitatively. To avoid issues from cut‐off choice altogether, we used the distribution of *P*_ST_ values instead when possible.

Organs differed in the proportion of differentially expressed genes between species. The most divergent organs are predominantly involved in interactions with the environment by detoxifying metabolites (kidney, liver) or by forming a direct contact of the inner body with the environment (lung). This may suggest that observed differences are at least in part due to regulatory plasticity rather than the fixation of regulatory variants (Gilad 2012; Romero *et al*. 2012). Organs that should be less affected by environmental effects, such as brain, showed markedly lower expression divergence and may provide an estimate of the base level of transcriptional divergence between species. Brain has been found to show a very low degree of expression divergence also in primates (Khaitovich *et al*. 2005; Lemos *et al*. 2005). Previous studies in flies (Nuzhdin *et al*. 2004) and primates (Khaitovich *et al*. 2005) have identified gene expression in testis as being highly divergent between species. A study in mice found qualitatively different, but not larger, gene expression divergence in testis compared to brain and liver (Bryk *et al*. 2013). In flycatchers, we found larger expression divergence, relative to expression variation, in testis than in all other organs, but lacked statistical power to identify a large number of differentially expressed genes due to small sample size for reproductive organs. Organs may differ for many other reasons than environmental impacts. The fact that the brain is a complex organ, built up of many different tissues and cell types, could affect the analysis and lead to underestimating the numbers of differentially expressed genes.

A comparison of our results obtained using the *P*_ST_ approach to those obtained using edgeR, an established method to detect differentially expressed genes, showed an overall agreement of methods. edgeR produced smaller numbers of differentially expressed genes, but this depends on the arbitrary choice of a significance cut‐off for detecting differentially expressed genes. When contrasting a set of differentially expressed genes with other genes, we chose the cut‐off such that the tested set of genes was large enough to have sufficient statistical power for tests. To minimize the impact of cut‐off choice, we used the distribution of *P*_ST_ values of all genes instead of a contrast whenever possible.

Analyses of the relationship between *P*_ST_ variance components and expression breadth (which is the inverse of organ specificity, i.e. 1‐τ) as well as the number of protein–protein interaction partners revealed negative correlations with within‐species expression variance, while correlations with between‐species variance were inconsistent or absent. This indicates that pleiotropy (for which both expression breadth and interactivity can be taken as proxies) constrains plasticity and/or genetic variability of gene expression within species, but does not necessarily hinder divergence between species, in accordance with theoretical models of gene expression evolution (Tulchinsky *et al*. 2014a,b). This was also true when comparing over the larger evolutionary distance to chicken, showing that this result was not due to an insufficient amount of expression divergence between the two flycatchers. Just as protein networks are sensitive to gene dosage (Papp *et al*. 2003), coregulated expression levels of network partners are likely to be critical for proper network or pathway function. However, the evolution of gene expression levels appears not to be impacted significantly by pleiotropic effects.

Divergence of gene expression levels should be associated with genetic changes in regulatory sequences, leading to the prediction of a correlation between gene expression and regulatory sequence divergence. We found no correlation between expression *P*_ST_ and genomic *F*_ST_ in 2‐kb regions upstream of the translation start site of genes. There are several possible explanations for this lack of correlation, including that the number of substitutions (or segregating variants with distinctly different allele frequencies between species) in regulatory sequences are too few to have a significant effect on *F*_ST_. Also, causative regulatory sites may not reside within the investigated 2‐kb intervals and expression divergence may at least partly be due to changes in trans‐acting factors. Renaut *et al*. (2012) similarly failed to find a correlation between genetic and gene expression divergence in lake whitefish and Bryk *et al*. (2013) did not find a significant overlap between differentially genes in genomic regions of selective sweeps and differentially expressed genes in house mice. We observed a positive correlation between *d*_N_/*d*_S_ and within‐species gene expression variance, but not between‐species variance or *P*_ST_, which could potentially reflect reduced constraint on both expression level and protein sequence of some genes.

An increasing body of evidence indicates that structural genomic variation, such as inversions, deletions or duplications, underlies many phenotypic differences within as well as between species (Mills *et al*. 2011; Gamazon & Stranger 2015). Based on the absence of expression from the *DPP7* gene in pied flycatchers and corroborated by a lack of reads mapping to the gene in genomic resequencing, we identified a ≈ 20‐kb deletion including most of *DPP7* in this species. DPP7 is a post‐proline cleaving aminopeptidase, widespread across vertebrate genomes, that is responsible for maintaining quiescence in T lymphocytes; its down‐regulation in T cells is associated with hyperproliferation in vivo (Mele *et al*. 2011). Mouse knockout mutants are embryonic lethal (Mele *et al*. 2011), which makes it surprising that such a gene is nonfunctional in a vertebrate species and that the deletion has apparently become fixed in pied flycatchers. Lineage‐sorting between collared flycatcher and pied flycatcher is far from complete. A recent study found 38% of 19.3 million SNPs identified in the two species from resequencing of 79 individuals per species from several populations to be shared (Nater *et al*. 2015). In the light of this recent divergence, rapid fixation of what intuitively would seem like a strongly deleterious mutation is unexpected. Perhaps linkage to an unrelated advantageous mutation has facilitated the spread of the haplotype carrying the *DPP7* deletion or that a loss of this gene for some reason has become bearable in pied flycatcher.

More than 12 000 expressed genes were detected per organ and species. Only 7.1% of the RNA‐seq reads mapped onto gene models, which is somewhat surprising but may be explained by that many RNAs are the result of leaky transcription (Kapranov *et al*. 2002; Johnson *et al*. 2005) and due to the rather conservative gene annotation primarily derived from sequence similarities to known genes in more or less closely related species. Annotation pipelines with more inclusive use of RNA‐seq evidence have produced higher gene numbers in related avian taxa (Poelstra *et al*. 2014) and efforts to improve on avian gene annotations using RNA‐seq evidence are on their way (Schmid *et al*. 2015). In any case, our study is likely to cover a significant proportion of protein‐coding genes in the two flycatcher species.

A major caveat in this study as in other studies of gene expression evolution is the difficulty of distinguishing between genetically mediated changes in expression and environmental effects (Gilad 2012; Romero *et al*. 2012; Meier *et al*. 2014; Morris *et al*. 2014). For within‐species variation, we sought to reduce environmental effects by collecting samples from the same breeding locality and at the same time point. Samples from the two species originated from different geographical areas but were collected at the same time point during the breeding period. For embryos, environmental influences should have been kept at a minimum due to the fact that eggs of both species were collected shortly after laying and then artificially incubated under identical conditions. Yet, there might be maternal effects adding nongenetic variance to embryonic expression levels. Studies of gene expression evolution in wild vertebrate populations are indeed associated with challenges when it comes to controlling for environmental influences.

H.E. conceived, designed and supervised the project; N.B. collected and processed samples; H.M. extracted RNA and constructed sequencing libraries; B.A., P.F., F.J.M. and S.M.J.S. generated gene annotations; S.U. analysed the data with input from A.K.; R.B. and C.F.M. provided statistical advice; P.B., R.B., L.D. and A.N. provided additional data; S.U. and H.E. wrote the manuscript with input from the other authors.

## Data accessibility

Sequence data: NCBI SRA, ERP001377.

## Supporting information


**Appendix S1** Genome annotation methods.
**Figure S1**. Expression variance (a), divergence (calculated as difference in log_2_ means) (b), and divergence/variance (c) compared for the different analyzed organs.
**Figure S2**. Outlier genes show both within‐ (σ_w_) and between‐species (σ_b_) variance components which spread over their whole respective genome‐wide ranges, except for small between‐species variance values (which is expected).
**Figure S3**. Proportion of outlier *P*_ST_ genes versus chromosome length per organ.
**Table S1**. Numbers of genes expressed in the different sequenced organs of collared and pied flycatchers.
**Table S2**. anova for the NMDS axes for collared flycatcher, performed with three axes.
**Table S3.** anova for the NMDS axes for collared and pied flycatcher together, which has been performed with four axes.
**Table S4.** anova for the NMDS axes for the two flycatchers and chicken, which has been performed with four axes.
**Table S5**. Correlation of gene expression variance between organs.
**Table S6**. GO terms enriched among genes with low or high expression variance, respectively.
**Table S7**. Proportion of differentially expressed genes in different tissues in relation to expression level threshold (cutoff).
**Table S8**. GO categories enriched among genes identified as differentially expressed using *P*_ST_ and edgeR.
**Table S9**. Correlations of *P*_ST_ and its variance components between collared and pied flycatcher with *d*_N_/*d*_S_.
**Table S10**. Correlations between *P*_ST_, and its variance components, for collared flycatcher‐chicken and proxies for pleiotropy.
**Table S11**. Genes expressed uniquely in collared flycatchers.
**Table S12**. Genes expressed uniquely in pied flycatchers.
