# Supplementary material for: Divergence in gene expression within and between two closely related flycatcher species
Source: Mol Ecol. 2016 Apr 2;25(9):2015–28. doi: 10.1111/mec.13596 (PMC4879514; doi:10.1111/mec.13596)
Supplement: Supplementary file 1 — Appendix S1 Genome annotation methods. Figure S1. Expression variance (a), divergence (calculated as difference in log2 means) (b), and divergence/variance (c) compared for the different analyzed organs. Figure S2. Outlier genes show both within‐ (σw) and between‐species (σb) variance components which spread over their whole respective genome‐wide ranges, except for small between‐species variance values (which is expected). Figure S3. Proportion of outlier P ST genes versus chromosome length per organ. Table S1. Numbers of genes expressed in the different sequenced organs of collared and pied flycatchers. Table S2. anova for the NMDS axes for collared flycatcher, performed with three axes. Table S3. anova for the NMDS axes for collared and pied flycatcher together, which has been performed with four axes. Table S4. anova for the NMDS axes for the two flycatchers and chicken, which has been performed with four axes. Table S5. Correlation of gene expression variance between organs. Table S6. GO terms enriched among genes with low or high expression variance, respectively. Table S7. Proportion of differentially expressed genes in different tissues in relation to expression level threshold (cutoff). Table S8. GO categories enriched among genes identified as differentially expressed using P ST and edgeR. Table S9. Correlations of P ST and its variance components between collared and pied flycatcher with d N/d S. Table S10. Correlations between P ST, and its variance components, for collared flycatcher‐chicken and proxies for pleiotropy. Table S11. Genes expressed uniquely in collared flycatchers. Table S12. Genes expressed uniquely in pied flycatchers. [file MEC-25-2015-s001.pdf]

Supplementary Information

Uebbing et al. Divergence in gene expression within and between two closely related flycatcher species

Supplementary Text

Repeat and simple feature annotation:

After loading into an Ensembl core schema database, the genomic sequence was screened for sequence patterns including repeats using RepeatMasker (version 3.2.8 with parameters '-nolow -species "aves" –s'), a custom RepeatModeler library we generated (version open-1.0.5), Dust and TRF. The RepeatMasker aves library and custom RepeatModeler library combined to mask 6.6% of the flycatcher genome.

Transcription start sites were predicted using Eponine–scan (Down & Hubbard 2002) and FirstEF (Davuluri *et al.* 2001). CpG islands longer than 400 bases (Micklem G, unpublished) and tRNAs (Lowe & Eddy 1997) were also predicted. The results of Eponine-scan, FirstEF, CpG, and tRNAscan are for display purposes only; they are not used in the gene annotation process. Genscan (Burge & Karlin1997) was run across repeat-masked sequence and the results were used as input for UniProt, UniGene (Sayers *et al.* 2010) and Vertebrate RNA (http://www.ebi.ac.uk/ena/) alignments by BLAST. Passing only Genscan results to BLAST is an effective way of reducing the search space and therefore the computational resources required. This resulted in 14,397,460 UniProt, 11,736,241 UniGene and 11,453,693 Vertebrate RNA sequences aligning to the genome.

Model generation

The gene annotation system is evidence-based; all protein coding and non-coding RNA gene models are supported by biological sequences from public databases. Input data for the protein coding gene models came from UniProt, data generated in this study and the Ensembl release 71 and 68 databases for chicken and zebra finch respectively. Data from each source were aligned to the genome and filtered in order to generate gene models.

Homology pipeline

Coding models were generated using data from related species. The genomic positions of the BLAST hits against the UniProt database were passed to GeneWise to build protein-coding models. This allowed GeneWise to run over a much reduced search space and create a slice-aware alignment of each target protein on the genome. This step generated a total of 194,425 initial gene models.

In addition, the longest protein coding translation was retrieved for each Ensembl gene for chicken (e71) and zebra finch (e68). These sequences were aligned to the flycatcher genome using Exonerate, generating 32,060 models. As Exonerate is a fast, splice-aware it was possible to run the entire set proteins across the genome, selecting the 'best in genome alignment' in each case. This method provided a useful alternative strategy to GeneWise.

RNA-seq pipeline

RNA-seq data used in the annotation comprised of paired end data from samples including: brain, embryo, kidney, liver, lung, muscle, ovary, skin, testis and a merged set of all nine organs from a number of individuals. The available reads were aligned to the genome using BWA. The Ensembl RNA-seq pipeline was used to process the BWA alignments and create further split read alignments using Exonerate. The RNA-seq pipeline produced 174,504 gene models in total. The predicted open reading frames were compared to UniProt Protein Existence (PE) classification level 1 and 2 proteins using BLAST. Models with poorly scoring or no BLAST alignments were split into a separate class

and not used in the final gene set.

Final protein-coding geneset creation:

The models produced by the RNA-seq and homology pipelines were filtered to select the highest quality models at each genomic position. Highest preference was given to RNA-seq models that were verified by a protein alignment covering 80-100 percent of the candidate ORF and with a percent identity of >= 80. Equal preference was given to models built from the homology pipeline based on bird proteins from SwissProt with PE level 1 or 2. Models from RNA-seq with poorer protein alignments, non-bird vertebrate models and models coming from Ensembl longest translation alignments were given lower precedence whenever a conflict in model structure occurred at a genomic position. Using this system the lower quality or redundant models were filtered out and a final geneset was created. UTR was added whenever possible using information from overlapping RNA-seq models.

References

Burge C, Karlin S (1997) Prediction of complete gene structures in human genomic DNA. *Journal of Molecular Biology*, **268:**78-94.

Davuluri RV, Grosse I, Zhang MQ (2001) Computational identification of promoters and first exons in the human genome. *Nature Genetics* 2001, **29:**412-417.

Down TA, Hubbard TJ (2002) Computational detection and location of transcription start sites in mammalian genomic DNA. *Genome Research* **12:**458-461.

Hart T, Komori HK, LaMere S, Podshivalova K, Salomon DR (2013) Finding the active genes in deep RNA-seq gene expression studies. *BMC Genomics*, **14:**778.

Lowe TM, Eddy SR(1997) tRNAscan-SE: a program for improved detection of transfer RNA genes in genomic sequence. *Nucleic Acids Research*, **25:**955-64.

Sayers EW, Barrett T, Benson DA *et al.* (2010) Database resources of the National Center for Biotechnology Information. *Nucleic Acids Research*, **38:**D5-16.

Table S1. Numbers of genes expressed in the different sequenced organs of collared and pied flycatchers.

**Organ**	**Collared Flycatcher**		**Pied Flycatcher**	
	**Expressed**	**Specific**	**Expressed**	**Specific**
Brain	12,855	439	13,051	635
Kidney	12,790	345	13,445	1,000
Liver	12,569	966	12,052	449
Lung	13,019	567	12,946	494
Muscle	12,616	823	12,348	555
Skin	13,232	373	13,783	924
Ovary	13,871	1,116	13,091	336
Testis	13,625	582	13,628	585
Embryo	13,723	488	13,816	581
All organs	15,118	149	15,099	129

Table S2. ANOVA for the NMDS axes for collared flycatcher, performed with three axes. Note that *p* values are Benjamini-Hochberg corrected for multiple testing.

	NMDS 1		NMDS 2		NMDS 3	
**Organ**	$F_{8,55} = 2238$	$p < 2.2 \cdot 10^{-16}$	$F_{8,55} = 406$	$p < 2.2 \cdot 10^{-16}$	$F_{8,55} = 430$	$p < 2.2 \cdot 10^{-16}$
**Sex**	$F_{1,55} = 0.408$	n.s.	$F_{1,55} = 1.28$	n.s.	$F_{1,55} = 0.068$	n.s.
**Organ*Sex**	$F_{6,55} = 4.78$	$p = 1.2 \cdot 10^{-3}$	$F_{6,55} = 1.46$	n.s.	$F_{6,55} = 0.358$	n.s.

Table S3. ANOVA for the NMDS axes for collared and pied flycatcher together, which has been performed with four axes. Note that *p* values are Benjamini-Hochberg corrected for multiple testing.

	**NMDS 1**		**NMDS 2**		**NMDS 3**		**NMDS 4**	
**Organ**	$F_{8,117}$ = 1360	$p < 2.2 \cdot 10^{-16}$	$F_{8,117}$ = 738	$p < 2.2 \cdot 10^{-16}$	$F_{8,117}$ = 957	$p < 2.2 \cdot 10^{-16}$	$F_{8,117}$ = 833	$p < 2.2 \cdot 10^{-16}$
**Species**	$F_{1,117}$ = 0.564	n.s.	$F_{1,117}$ = 0.078	n.s.	$F_{1,117}$ = 16.1	$p = 4.9 \cdot 10^{-4}$	$F_{1,117}$ = 0.124	n.s.
**Sex**	$F_{1,117}$ = 3.41	n.s.	$F_{1,117}$ = 6.50	$p = 2.8 \cdot 10^{-2}$	$F_{1,117}$ = 0.007	n.s.	$F_{1,117}$ = 10.4	$p = 6.6 \cdot 10^{-3}$
**Organ*Species**	$F_{8,117}$ = 6.52	$p = 2.9 \cdot 10^{-6}$	$F_{8,117}$ = 1.94	n.s.	$F_{8,117}$ = 3.21	$p = 8.6 \cdot 10^{-3}$	$F_{8,117}$ = 0.706	n.s.
**Organ*Sex**	$F_{6,117}$ = 3.19	$p = 1.7 \cdot 10^{-2}$	$F_{6,117}$ = 1.91	n.s.	$F_{6,117}$ = 0.524	n.s.	$F_{6,117}$ = 2.76	$p = 3.3 \cdot 10^{-2}$
**Species* Sex**	$F_{1,117}$ = 6.38	$p = 2.6 \cdot 10^{-2}$	$F_{1,117}$ = 1.01	n.s.	$F_{1,117}$ = 0.420	n.s.	$F_{1,117}$ = 0.946	n.s.
**Organ*Species*Sex**	$F_{6,117}$ = 3.36	$p = 1.4 \cdot 10^{-2}$	$F_{6,117}$ = 0.855	n.s.	$F_{6,117}$ = 0.084	n.s.	$F_{6,117}$ = 2.43	n.s.

Table S4. ANOVA for the NMDS axes for the two flycatchers and chicken, which has been performed with four axes. Note that *p* values are Benjamini-Hochberg corrected for multiple testing.

	**NMDS 1**		**NMDS 2**		**NMDS 3**		**NMDS 4**	
**Organ**	$F_{6,109} = 3162$	$p < 2.2 \cdot 10^{-16}$	$F_{6,109} = 1731$	$p < 2.2 \cdot 10^{-16}$	$F_{6,109} = 815$	$p < 2.2 \cdot 10^{-16}$	$F_{6,109} = 952$	$p < 2.2 \cdot 10^{-16}$
**Species**	$F_{2,109} = 153$	$p < 2.2 \cdot 10^{-16}$	$F_{2,109} = 108$	$p < 2.2 \cdot 10^{-16}$	$F_{2,109} = 383$	$p < 2.2 \cdot 10^{-16}$	$F_{2,109} = 821$	$p < 2.2 \cdot 10^{-16}$
**Sex**	$F_{1,109} = 1.30$	n.s.	$F_{1,109} = 3.56$	n.s.	$F_{1,109} = 2.14$	n.s.	$F_{1,109} = 16.3$	$p = 2.2 \cdot 10^{-4}$
**Organ*Species**	$F_{12,109} = 9.80$	$p = 2.9 \cdot 10^{-12}$	$F_{12,109} = 4.84$	$p = 5.8 \cdot 10^{-6}$	$F_{12,109} = 15.1$	$p < 2.2 \cdot 10^{-16}$	$F_{12,109} = 8.57$	$p = 7.5 \cdot 10^{-11}$
**Organ*Sex**	$F_{4,109} = 0.242$	n.s.	$F_{4,109} = 2.68$	n.s.	$F_{4,109} = 1.29$	n.s.	$F_{4,109} = 2.10$	n.s.
**Species*Sex**	$F_{2,109} = 0.981$	n.s.	$F_{2,109} = 0.310$	n.s.	$F_{2,109} = 1.52$	n.s.	$F_{2,109} = 1.09$	n.s.
**Organ*Species*Sex**	$F_{8,109} = 0.692$	n.s.	$F_{8,109} = 0.654$	n.s.	$F_{8,109} = 0.219$	n.s.	$F_{8,109} = 0.694$	n.s.

Table S5. Correlation of gene expression variance between organs. The lower left half shows Spearman's ρ between collared flycatcher organs, the upper right half between pied flycatcher organs. The diagonal (italics) shows correlations in the same organ between species. All comparisons were at $p < 2.2 \cdot 10^{-16}$ after Benjamini-Hochberg correction.

**Organ**	**Brain**	**Kidney**	**Liver**	**Lung**	**Muscle**	**Skin**	**Ovary**	**Testis**	**Embryo**
Brain	*0.626*	0.334	0.242	0.356	0.325	0.220	0.197	0.294	0.268
Kidney	0.318	*0.491*	0.336	0.390	0.320	0.375	0.247	0.286	0.271
Liver	0.295	0.410	*0.488*	0.291	0.357	0.280	0.148	0.164	0.179
Lung	0.335	0.380	0.254	*0.527*	0.341	0.476	0.304	0.291	0.301
Muscle	0.312	0.414	0.382	0.300	*0.537*	0.337	0.213	0.230	0.276
Skin	0.246	0.393	0.281	0.384	0.270	*0.502*	0.277	0.271	0.323
Ovary	0.282	0.271	0.236	0.259	0.246	0.220	*0.285*	0.225	0.243
Testis	0.231	0.243	0.233	0.234	0.247	0.226	0.219	*0.396*	0.288
Embryo	0.332	0.332	0.272	0.327	0.303	0.311	0.249	0.254	*0.342*

Table S6. GO terms enriched among genes with low or high expression variance, respectively. *P* values are Bonferroni corrected.

**Ontology**	**GO term**	**Low or high variance genes/total genes**	***p* value**
		**Low expression variance**	
MF	G-protein coupled receptor activity	68/318	$1.86 \cdot 10^{-7}$
MF	signal transducer activity	76/399	$3.56 \cdot 10^{-6}$
BP	G-protein coupled receptor signaling pathway	74/394	$1.24 \cdot 10^{-5}$
BP	response to stimulus	114769/	$6.88 \cdot 10^{-4}$
MF	poly(A) RNA binding	12/21	$7.86 \cdot 10^{-4}$
		**High expression variance**	
CC	extracellular region	93/370	$7.97 \cdot 10^{-18}$
CC	extracellular space	101490/	$1.69 \cdot 10^{-13}$
CC	blood microparticle	26/53	$3.84 \cdot 10^{-10}$
MF	heme binding	33/99	$4.47 \cdot 10^{-8}$
MF	calcium ion binding	80/435	$9.30 \cdot 10^{-7}$
BP	negative regulation of endopeptidase activity	22/59	$1.81 \cdot 10^{-5}$
CC	intermediate filament	21/56	$3.78 \cdot 10^{-5}$
CC	myofibril	27/97	$4.53 \cdot 10^{-4}$
MF	structural molecule activity	11/18	$1.63 \cdot 10^{-3}$
MF	monooxygenase activity	18/54	$4.08 \cdot 10^{-3}$
CC	troponin complex	6/6	$5.26 \cdot 10^{-3}$
MF	lipid binding	18/57	$1.02 \cdot 10^{-2}$
MF	transporter activity	32/153	$2.81 \cdot 10^{-2}$
MF	hormone activity	6/7	$3.04 \cdot 10^{-2}$
CC	high-density lipoprotein particle	14/39	$3.41 \cdot 10^{-2}$
MF	structural constituent of muscle	17/56	$4.32 \cdot 10^{-2}$
MF	cysteine-type endopeptidase inhibitor activity	7/10	$4.32 \cdot 10^{-2}$
BP	muscle contraction	9/17	$4.34 \cdot 10^{-2}$

Table S7. Proportion of differentially expressed genes in different tissues in relation to expression level threshold (cutoff). Genes with low expression levels have low power in statistical testing, hence the negative correlation beween the proportion of differentially expressed genes and cutoff. Reported amounts of differentially expressed in the main text are based on analyses with a lower cutoff of 0.125 as suggested by Hart *et al*. (2013).

**Organ**	**Expression level cutoff (zFPKM)**				
	**0.125 (~detection level)**	**1.0**	**2.0**	**4.0**	**8.0**
Brain	0.024	0.035	0.057	0.092	0.121
Kidney	0.142	0.184	0.231	0.273	0.305
Liver	0.264	0.317	0.358	0.400	0.405
Lung	0.183	0.211	0.230	0.265	0.287
Muscle	0.064	0.083	0.111	0.151	0.175
Skin	0.089	0.117	0.158	0.212	0.242
Ovary	0.012	0.016	0.019	0.022	0.027
Testis	0.005	0.006	0.007	0.011	0.019
Embryo	0.086	0.105	0.134	0.161	0.195

Table S8. GO categories enriched among genes identified as differentially expressed using P_{ST} and EDGER.

Organ	GO terms	*p* (P_{ST})	*p* (edgeR)
Brain	-		
Kidney	mitochondrion (CC)	$8.35 \cdot 10^{-5}$	$1.15 \cdot 10^{-9}$
	extracellular vesicular exosome (CC)	n.s.	$5.48 \cdot 10^{-6}$
	oxidation-reduction process (BP)	n.s.	$7.83 \cdot 10^{-5}$
	oxidoreductase activity (MF)	n.s.	$5.54 \cdot 10^{-4}$
Liver	poly(A) RNA binding (MF)	$3.81 \cdot 10^{-11}$	$7.32 \cdot 10^{-7}$
	mitochondrion (CC)	$4.45 \cdot 10^{-6}$	$1.70 \cdot 10^{-16}$
	mitochondrial inner membrane (CC)	$6.74 \cdot 10^{-5}$	$5.70 \cdot 10^{-7}$
	extracellular vesicular exosome (CC)	$4.03 \cdot 10^{-2}$	$5.07 \cdot 10^{-5}$
	ribonucleoprotein complex (CC)	n.s.	$1.93 \cdot 10^{-10}$
	structural constituent of ribosome (MF)	n.s.	$6.77 \cdot 10^{-8}$
	oxidation-reduction process (BP)	n.s.	$1.16 \cdot 10^{-7}$
	ribosome (CC)	n.s.	$2.04 \cdot 10^{-7}$
	translation (BP)	n.s.	$1.03 \cdot 10^{-6}$
	oxidoreductase activity (MF)	n.s.	$9.93 \cdot 10^{-4}$
	mitochondrial respiratory chain complex I (CC)	n.s.	$1.08 \cdot 10^{-2}$
	cellular response to arsenic-containing substance (BP)	n.s.	$1.54 \cdot 10^{-2}$
Lung	poly(A) RNA binding (MF)	$1.80 \cdot 10^{-7}$	n.s.
	ribonucleoprotein complex (CC)	$1.78 \cdot 10^{-2}$	n.s.
	GDP binding (MF)	$3.05 \cdot 10^{-2}$	n.s.
Muscle	-		
Skin	poly(A) RNA binding (MF)	$5.07 \cdot 10^{-13}$	n.s.
	ribonucleoprotein complex (CC)	$6.82 \cdot 10^{-7}$	n.s.
	translation (BP)	$1.14 \cdot 10^{-4}$	n.s.
	structural constituent of ribosome (MF)	$3.61 \cdot 10^{-4}$	n.s.
	ribosome (CC)	$3.77 \cdot 10^{-4}$	n.s.
	cytosolic large ribosomal subunit (CC)	$4.56 \cdot 10^{-3}$	n.s.
	nucleosome assembly (BP)	$4.57 \cdot 10^{-2}$	n.s.
	extracellular vesicular exosome (CC)	n.s.	$3.13 \cdot 10^{-5}$
Ovary	hyaluronan metabolic process (BP)	n.s.	$4.12 \cdot 10^{-2}$
Testis	-		

Table S9. Correlations of P_{ST} and its variance components between collared and pied flycatcher with d_N/d_S.

**Organ**	P_{ST}		**Between-species variance (σ_b)**		**Within-species variance (σ_w)**	
	**ρ**	***p* value**	**ρ**	***p* value**	**ρ**	***p* value**
Brain	-0.040	0.027	-0.043	0.012	0.191	$< 2.2 \cdot 10^{-16}$
Kidney	-0.015	0.46	-0.016	0.41	0.131	$< 2.2 \cdot 10^{-16}$
Liver	-0.009	0.67	0.023	0.26	0.145	$< 2.2 \cdot 10^{-16}$
Lung	-0.027	0.15	-0.027	0.13	0.208	$< 2.2 \cdot 10^{-16}$
Muscle	0.013	0.55	0.036	0.055	0.124	$5.6 \cdot 10^{-13}$
Skin	-0.084	$6.4 \cdot 10^{-7}$	-0.060	$2.0 \cdot 10^{-4}$	0.201	$< 2.2 \cdot 10^{-16}$
Ovary	0.001	0.94	-0.014	0.46	0.107	$2.1 \cdot 10^{-11}$
Testis	-0.002	0.94	-0.010	0.58	0.071	$4.2 \cdot 10^{-6}$
Embryo	-0.076	$6.6 \cdot 10^{-6}$	-0.043	0.010	0.132	$< 2.2 \cdot 10^{-16}$

Table S10. Correlations between P_{ST}, and its variance components, for collared flycatcher-chicken and proxies for pleiotropy.

Organ	P_{ST}		Between-species variance (σ_b)		Within-species variance (σ_w)	
	**Metabolic chains**					
Brain	ρ = 0.113	$p < 2.2 \cdot 10^{-16}$	ρ = 0.057	$p = 1.4 \cdot 10^{-5}$	ρ = -0.223	$p < 2.2 \cdot 10^{-16}$
Kidney	ρ = 0.045	$p = 3.8 \cdot 10^{-4}$	ρ = -0.021	$p = 0.16$	ρ = -0.239	$p < 2.2 \cdot 10^{-16}$
Liver	ρ = 0.030	$p = 0.035$	ρ = -0.044	$p = 0.0026$	ρ = -0.224	$p < 2.2 \cdot 10^{-16}$
Ovary	ρ = 0.042	$p = 8.6 \cdot 10^{-4}$	ρ = 0.053	$p = 4.1 \cdot 10^{-5}$	ρ = -0.151	$p < 2.2 \cdot 10^{-16}$
Testis	ρ = 0.067	$p = 5.3 \cdot 10^{-8}$	ρ = 0.016	$p = 0.28$	ρ = -0.214	$p < 2.2 \cdot 10^{-16}$
	**Protein complexes**					
Brain	ρ = 0.124	$p < 2.2 \cdot 10^{-16}$	ρ = 0.070	$p = 1.3 \cdot 10^{-7}$	ρ = -0.199	$p < 2.2 \cdot 10^{-16}$
Kidney	ρ = 0.044	$p = 4.9 \cdot 10^{-4}$	ρ = -0.009	$p = 0.49$	ρ = -0.208	$p < 2.2 \cdot 10^{-16}$
Liver	ρ = 0.027	$p = 0.055$	ρ = -0.034	$p = 0.026$	ρ = -0.184	$p < 2.2 \cdot 10^{-16}$
Ovary	ρ = 0.057	$p = 6.2 \cdot 10^{-6}$	ρ = 0.063	$p = 1.4 \cdot 10^{-6}$	ρ = -0.114	$p < 2.2 \cdot 10^{-16}$
Testis	ρ = 0.057	$p = 3.7 \cdot 10^{-6}$	ρ = 0.012	$p = 0.38$	ρ = -0.178	$p < 2.2 \cdot 10^{-16}$
	**Signaling cascades**					
Brain	ρ = 0.062	$p = 1.3 \cdot 10^{-6}$	ρ = 0.022	$p = 0.16$	ρ = -0.145	$p < 2.2 \cdot 10^{-16}$
Kidney	ρ = 0.021	$p = 0.087$	ρ = -0.014	$p = 0.31$	ρ = -0.145	$p < 2.2 \cdot 10^{-16}$
Liver	ρ = 0.024	$p = 0.087$	ρ = -0.020	$p = 0.23$	ρ = -0.138	$p < 2.2 \cdot 10^{-16}$
Ovary	ρ = 0.001	$p = 0.94$	ρ = 0.008	$p = 0.53$	ρ = -0.089	$p = 1.2 \cdot 10^{-13}$
Testis	ρ = 0.013	$p = 0.29$	ρ = -0.013	$p = 0.33$	ρ = -0.118	$p < 2.2 \cdot 10^{-16}$
	**Organ expression specifitcity (τ)**					
Brain	ρ = -0.031	$p = 0.014$	ρ = -0.006	$p = 0.57$	ρ = 0.090	$p = 1.6 \cdot 10^{-15}$
Kidney	ρ = 0.028	$p = 0.021$	ρ = 0.068	$p = 1.6 \cdot 10^{-9}$	ρ = 0.151	$p < 2.2 \cdot 10^{-16}$
Liver	ρ = 0.023	$p = 0.064$	ρ = 0.075	$p = 1.6 \cdot 10^{-9}$	ρ = 0.141	$p < 2.2 \cdot 10^{-16}$
Ovary	ρ = -0.023	$p = 0.049$	ρ = -0.030	$p = 0.0094$	ρ = 0.199	$p < 2.2 \cdot 10^{-16}$
Testis	ρ = 0.082	$p = 3.0 \cdot 10^{-13}$	ρ = 0.099	$p < 2.2 \cdot 10^{-16}$	ρ = 0.086	$p = 2.0 \cdot 10^{-15}$

Table S11. Genes expressed uniquely in collared flycatchers. Genes are counted if detected in at least five individuals in collared flycatchers, and none in pied flycatchers.

**Gene ID**	**Gene name**	**Chromosome**	**Expressed in**
ENSFALG00000003102	*novel*	26	brain, muscle, skin, ovary, embryo
ENSFALG00000003217	*novel*	25	liver, skin, ovary, embryo
ENSFALG00000004753	*DPP7*	17	all organs
ENSFALG00000014612	*novel*	2	kidney, liver, lung, muscle
ENSFALG00000015165	*novel*	1	liver, skin, gonads, embryo

Table S12. Genes expressed uniquely in pied flycatchers. Genes are counted if detected in at least five individuals in pied flycatchers, and none in collared flycatchers.

**Gene ID**	**Gene name**	**Chromosome**	**Expressed in**
ENSFALG00000000195	*VSIG1*	4A	lung, embryo
ENSFALG00000000703	*GCG*	7	testis, embryo
ENSFALG00000000849	*novel*	23	brain, kidney, skin, testis, embryo
ENSFALG00000003076	*CNGA1*	4	brain, liver, lung, muscle, skin, testis
ENSFALG00000004044	*OCA2*	1	brain, liver, skin, testis, embryo
ENSFALG00000004891	*novel*	1	lung, skin, ovary
ENSFALG00000010083	*IL22*	1A	brain, lung, muscle, skin, testis
ENSFALG00000010694	*novel*	20	brain, muscle
ENSFALG00000012860	*novel*	34	kidney
ENSFALG00000015446	*novel*	5	skin, testis
ENSFALG00000016024	*uc_338*	7	brain, lung, embryo

Figure legends

Figure S1. Expression variance (a), divergence (calculated as difference in log_2 means) (b), and divergence/variance (c) compared for the different analyzed organs. Boxes represent quartiles, whiskers the maximum of the distribution.

Figure S2. Outlier genes show both within- (σ_w) and between-species (σ_b) variance components which spread over their whole respective genome-wide ranges, except for small between-species variance values (which is expected). Outlier genes lie approximately above the diagonal through the origin with slope 1 (dashed line). The data shown is from skin as a representative example organ.

Figure S3. Proportion of outlier P_{ST} genes versus chromosome length per organ. The Z chromosome is shown as a filled circle.

Figure S4. Genes expressed uniquely in one of the species are likely to be lowly expressed. The plot shows data from brain as a representative example organ. n_{ind}, number of individuals of both species a gene was detected in; n_{genes}, number of genes expressed in that category; n_{uniq}, number of genes in that category which was uniquely expressed in one of the species. Boxes represent quartiles, whiskers the minimum of either 5x the interquartile range or the maximum of the distribution.

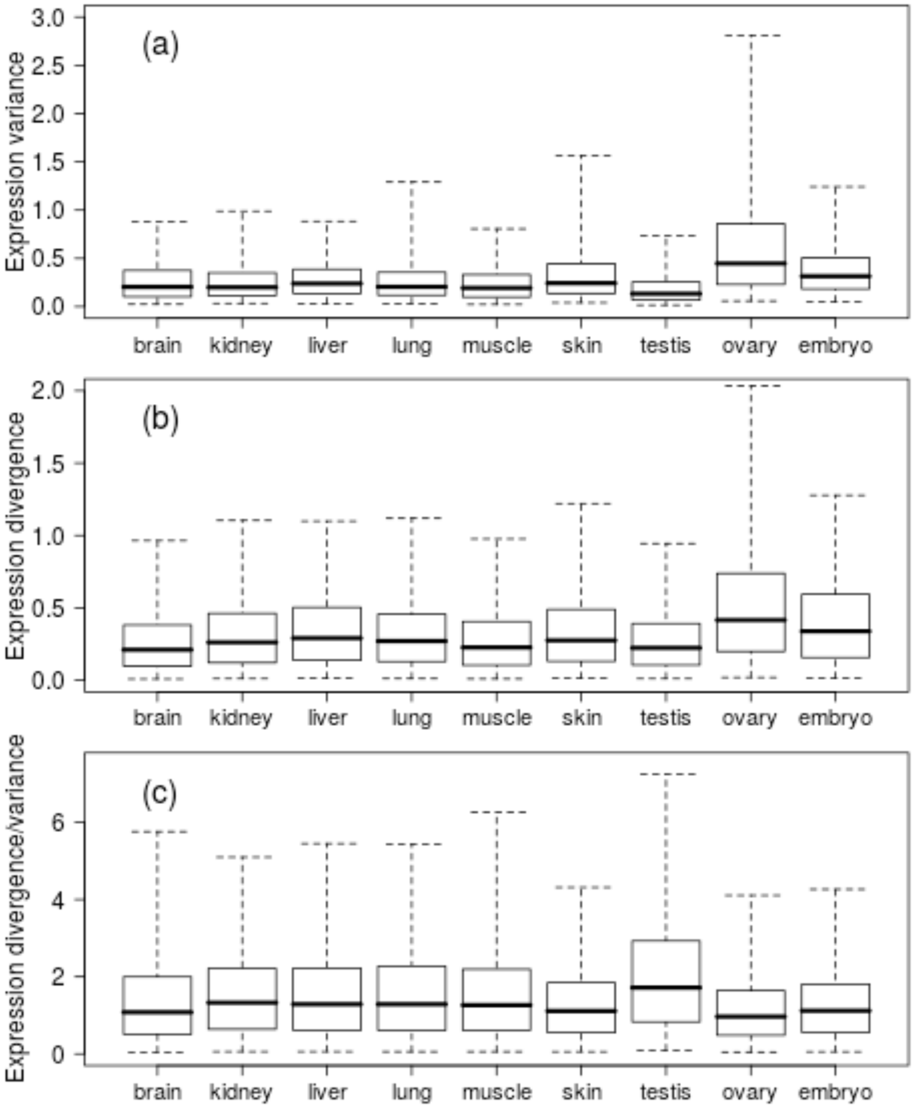
(a)
Expression variance
3.0
2.5
2.0
1.5
1.0
0.5
0.0
brain
kidney
liver
lung
muscle
skin
testis
ovary
embryo
(b)
Expression divergence
2.0
1.5
1.0
0.5
0.0
brain
kidney
liver
lung
muscle
skin
testis
ovary
embryo
(c)
Expression divergence/variance
6
4
2
0
brain
kidney
liver
lung
muscle
skin
testis
ovary
embryo

Figure S1

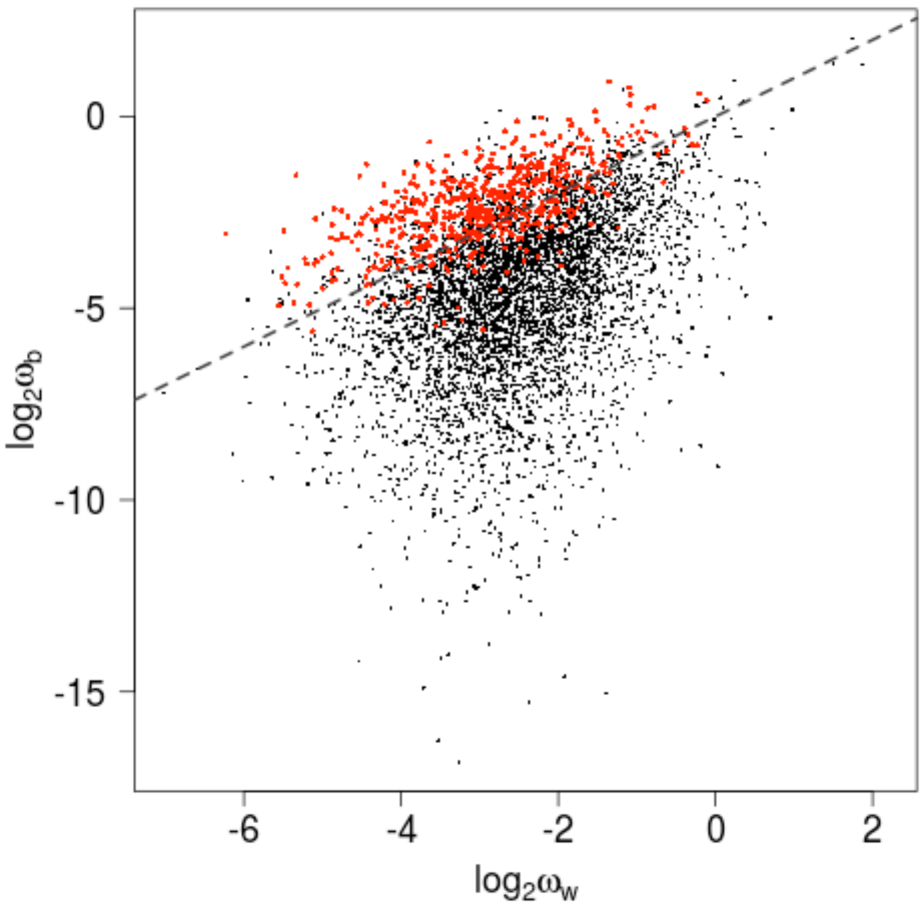

Figure S2

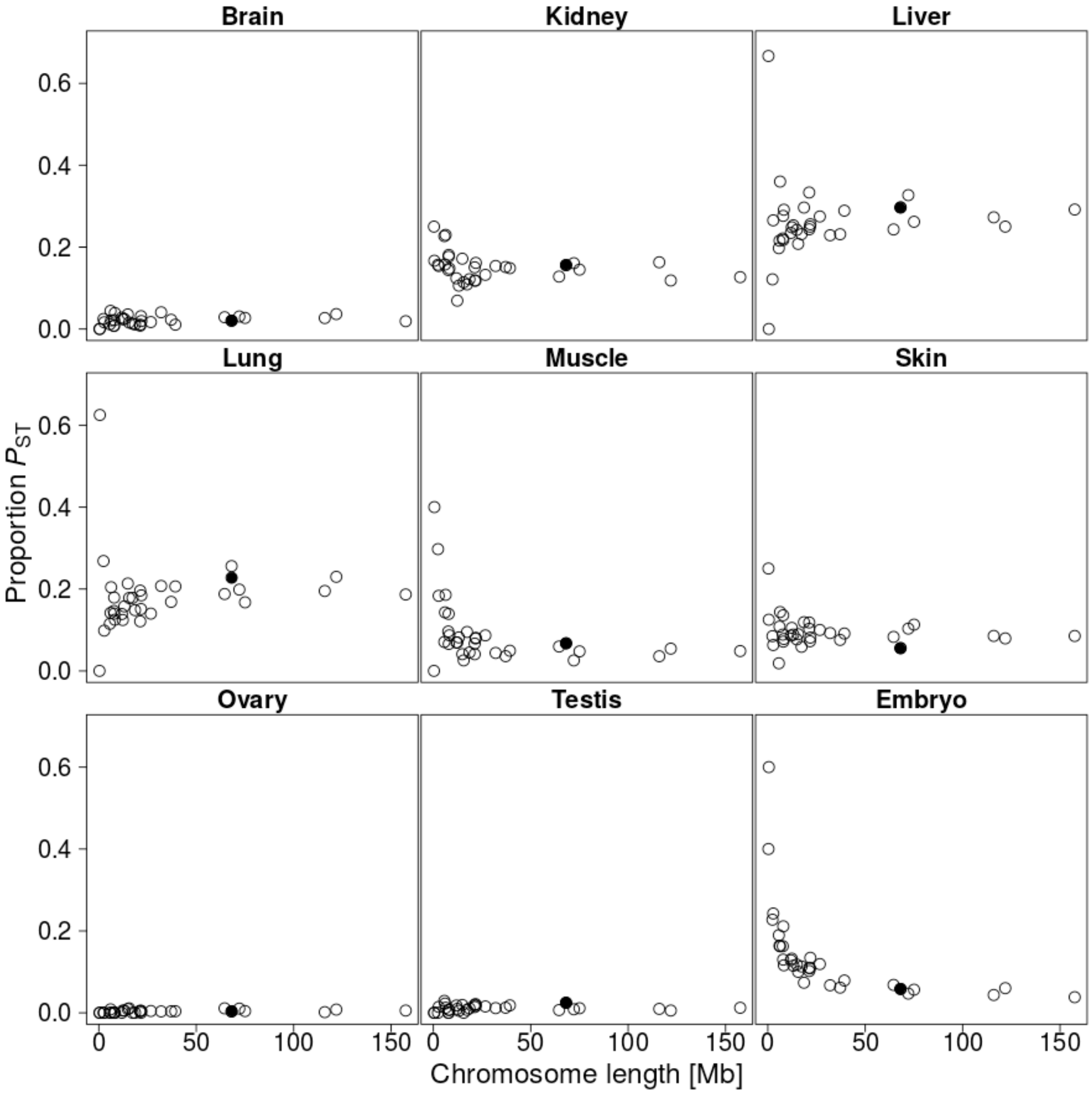

Brain
Kidney
Liver
Lung
Muscle
Skin
Ovary
Testis
Embryo
Proportion P_{ST}
0.0
0.2
0.4
0.6
0
50
100
150
Chromosome length [Mb]

Figure S3

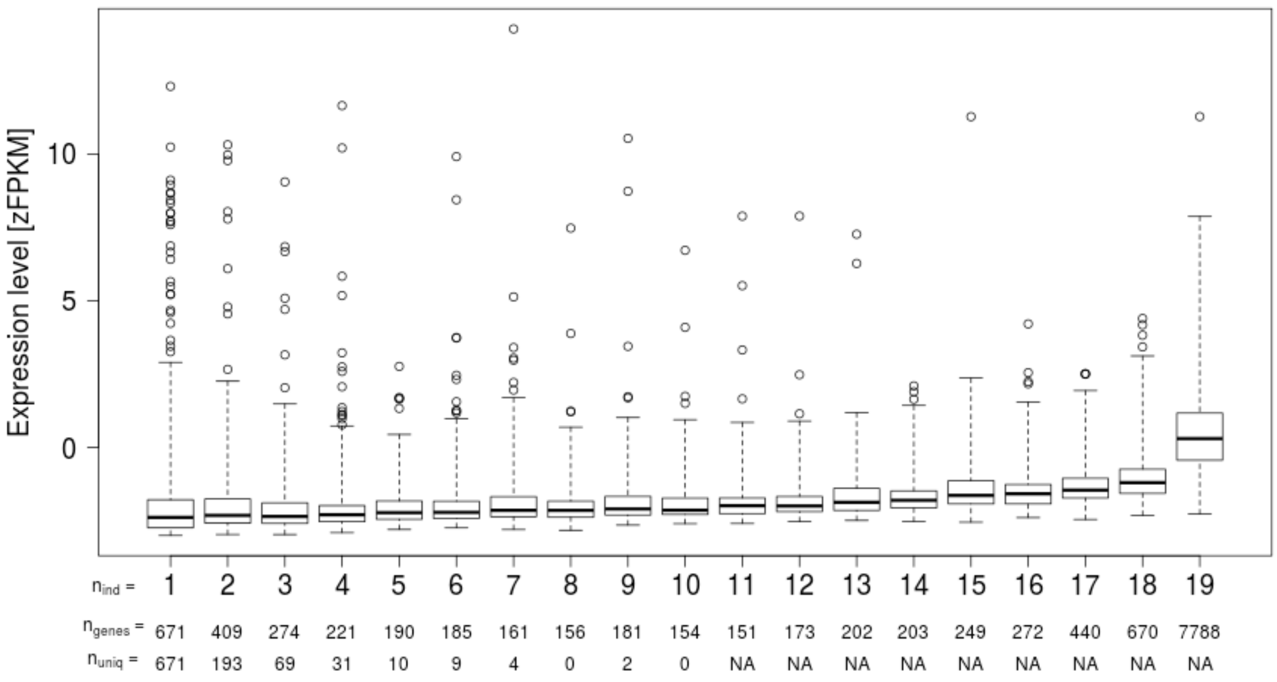
Expression level [zFPKM]
10
5
0
n_ind = 1 2 3 4 5 6 7 8 9 10 11 12 13 14 15 16 17 18 19
n_genes = 671 409 274 221 190 185 161 156 181 154 151 173 202 203 249 272 440 670 7788
n_uniq = 671 193 69 31 10 9 4 0 2 0 NA NA NA NA NA NA NA NA NA

Figure S4
